# Spike-Train Level Direct Feedback Alignment: Sidestepping Backpropagation for On-Chip Training of Spiking Neural Nets

**DOI:** 10.3389/fnins.2020.00143

**Published:** 2020-03-13

**Authors:** Jeongjun Lee, Renqian Zhang, Wenrui Zhang, Yu Liu, Peng Li

**Affiliations:** ^1^Department of Electrical and Computer Engineering, University of California, Santa Barbara, Santa Barbara, CA, United States; ^2^Department of Electrical and Computer Engineering, Texas A&M University, College Station, TX, United States

**Keywords:** spiking neural networks, backpropagation, on-chip training, hardware neural processor, FPGA

## Abstract

Spiking neural networks (SNNs) present a promising computing model and enable bio-plausible information processing and event-driven based ultra-low power neuromorphic hardware. However, training SNNs to reach the same performances of conventional deep artificial neural networks (ANNs), particularly with error backpropagation (BP) algorithms, poses a significant challenge due to inherent complex dynamics and non-differentiable spike activities of spiking neurons. In this paper, we present the first study on realizing competitive spike-train level backpropagation (BP) like algorithms to enable on-chip training of SNNs. We propose a novel spike-train level direct feedback alignment (ST-DFA) algorithm, which is much more bio-plausible and hardware friendly than BP. Algorithm and hardware co-optimization and efficient online neural signal computation are explored for on-chip implementation of ST-DFA. On the Xilinx ZC706 FPGA board, the proposed hardware-efficient ST-DFA shows excellent performance vs. overhead tradeoffs for real-world speech and image classification applications. SNN neural processors with on-chip ST-DFA training show competitive classification accuracy of 96.27% for the MNIST dataset with 4× input resolution reduction and 84.88% for the challenging 16-speaker TI46 speech corpus, respectively. Compared to the hardware implementation of the state-of-the-art BP algorithm HM2-BP, the design of the proposed ST-DFA reduces functional resources by 76.7% and backward training latency by 31.6% while gracefully trading off classification performance.

## 1. Introduction

As a brain-inspired computational model, spiking neural networks (SNNs) have gathered significant research interests during recent years. The spike-based operational principles of SNNs support a variety of information coding schemes including temporal codes and have rendered energy-efficient VLSI neuromorphic chips, such as IBM's TrueNorth (Akopyan et al., 2015) and Intel's Loihi (Davies et al., 2018). Despite the recent progresses in SNNs and neuromorphic processor designs, fully leveraging the theoretical computing advantages of SNNs over traditional artificial neural networks (ANNs) (Maass, 1997) to achieve the state-of-the-art performance for real-world applications remains challenging. One chief difficulty here lies in training of SNNs in terms of achievable performance and computational complexity.

In terms of the learning algorithm of SNNs, there are several algorithms, such as Spike-timing-dependent plasticity (STDP)/binary/probabilistic and error backpropagation (BP). Each algorithm has gathered significant research interests with the advantages of each algorithm. For example, STDP mimics biological behavior using the timing between pre- and post-synaptic spikes, and BP has shown the state-of-the-art performance in ANNs, implying its potential to be used in SNNs for achieving excellent accuracy. While the above algorithms provide a rich set of learning mechanisms that can be explored, as of now, SNNs exploiting a non-BP algorithm, such as a STDP/binary/probabilistic method have demonstrated limited success in competitive real-world applications. Recent advances in BP have provided algorithms for overcoming non-differentiability of spike events and capturing temporal dynamics, and have made it possible to achieve the state-of-the-art performances among many other algorithms.

Inspired by the success of BP and its variants, such as stochastic gradient decent in training conventional ANNs (Rumelhart et al., 1988a), various SNN BP methods have emerged, aiming at attaining the same level of performance (Bohte et al., 2002; Lee et al., 2016; Jin et al., 2018; Wu et al., 2018; Chankyu et al., 2019; Panda et al., 2019). The major challenges in BP training of SNNs stem from the non-differentiability of spike events and temporal dynamics that prevent straightforward derivative computation. SpikeProp (Bohte et al., 2002) is the first BP algorithm to train SNNs by BP. However, SpikeProp is limited to single-spike training for learning simple functions like XOR. Lee et al. (2016) proposes a BP algorithm which differentiates neuron's membrane potential instead of discrete output spikes. Wu et al. (2018) improves Lee et al. (2016) by capturing temporal effects with backpropagation through time (BPTT) (Werbos, 1990). However, the error gradient is still computed by differentiating the membrane potential, leading to inconsistency w.r.t the rate-coded loss function. More recently, Panda et al. (2019) provides the hybrid neural network architecture for approximate gradient descent (AGD) training methodology achieving good accuracy with large datasets, such as Imagenet, and Chankyu et al. (2019) proposes differentiable activation for leaky integrate-and-fire (LIF) spiking neurons using a spike-based BP algorithm achieving good classification accuracies with various datasets.

This paper is motivated by one of these approaches (i.e., Jin et al., 2018), which showed a spike-train level BP algorithm that achieves the state-of-the-art performance on SNNs. Jin et al. (2018) proposes a hybrid macro/micro level backpropagation (HM2-BP) algorithm for training multi-layer SNNs, which addresses the aforementioned issues. HM2-BP precisely captures the temporal behavior of the SNN at the microscopic level and directly computes the gradient of the rate-coded loss function w.r.t tunable parameters. As a result, HM2-BP demonstrates the state-of-the art learning performances on widely adopted SNN benchmarks, such as MNIST (LeCun et al., 1998) and Neuromorphic-MNIST (N-MNIST) (Orchard et al., 2015), outperforming all other existing BP algorithms based on the leaky integrate-and-fire model.

While achieving excellent results, the aforementioned SNN BP algorithms are hampered by several limitations. The error signal is propagated backward layer by layer through weights symmetric to the feed-forward weights. This is considered not biologically-plausible. Furthermore, BP algorithms involve complex layer-by-layer backward computations, which is expensive to implement on-chip and introduces high training latency. For instance, while HM2-BP improves the scalability of BPTT (Wu et al., 2018) by operating on the spike-train level, i.e., application of BP does not discretize time, it still involves complex computations and its latency in the backward phase is proportional to network depth.

This work aims to answer the following questions: (1) Can biologically plausible mechanisms be developed to sidestep complex BP algorithms while delivering competitive performance? (2) Can such mechanisms be leveraged for efficient on-chip training of multi-layer SNNs?

We are motivated by the recent direct feedback alignment (DFA) method developed for conventional ANNs (Nøkland, 2016), where the error is more biologically-plausibly fed back to each hidden layer through fixed random feedback connections directly from the output layer, reducing a bulk of the BP complexity. Furthermore, DFA can be performed for all hidden layers concurrently, reducing the backward phase latency.

By extending the DFA concept proposed by Nøkland (2016) for SNNs, we significantly reduce hardware overhead and latency of the network, while maintaining the advantage of a well-defined BP-like algorithm in terms of accuracy. Although many algorithms, such as gradient descent (GD), AGD, and BP, have been proposed for SNNs, this is the first work presenting algorithm-hardware co-optimization and demonstrating the realization of DFA for SNNs with significantly reduced hardware cost while maintaining competitive accuracies for image/speech recognition tasks. The main contributions of this work are:

- We demonstrate the *first* direct feedback alignment algorithm for training multi-layer SNNs by extending the DFA concept developed for conventional ANNs;
- Our spiking DFA algorithm is embodied at the spike-train level, dubbed ST-DFA, to further improve scalability by avoiding involved error feedback over time;
- We perform algorithm-hardware co-optimization and demonstrate the *first* hardware realization of DFA for SNNs with significantly reduced hardware overhead, energy dissipation, and latency while achieving competitive performances for image/speech recognition tasks.

On the Xilinx ZC706 FPGA board, the proposed ST-DFA with optimized implementation shows excellent cost-effectiveness for on-chip SNN training. Hardware SNNs with ST-DFA deliver competitive accuracy of 96.27% for the MNIST (LeCun et al., 1998) with 4× input resolution reduction and 84.88% for the challenging 16-speaker TI46 (Liberman et al., 1991) speech corpus, respectively. Compared to the hardware implementation of the state-of-the-art BP algorithm HM2-BP, the design of the proposed ST-DFA reduces functional resources by 76.7% and backward training latency by 31.6% while gracefully trading off classification performance.

## 2. Materials and Methods

### 2.1. Background

#### 2.1.1. Direct Feedback Alignment

Backpropagation (BP) has been widely applied to train neural networks. It is based upon computing a global error at the output layer and then propagating the error signal to hidden neurons layer by layer. During this process, the errors of a preceding layer are multiplied with a weight matrix that is completely symmetric to the one for the feed-forward connections. This fact is not considered biologically plausible. A recent discovery called Feedback Alignment (FA) (Lillicrap et al., 2016) demonstrates that the weights used for propagating the error layer by layer need not be symmetric to the weights used for forward propagation to achieve good performance. The feedback weight matrix can be randomly generated and then stay unchanged since the network can learn how to make feedback useful through training. Neftci et al. (2017) applies FA for training SNNs.

**Figure 1:**
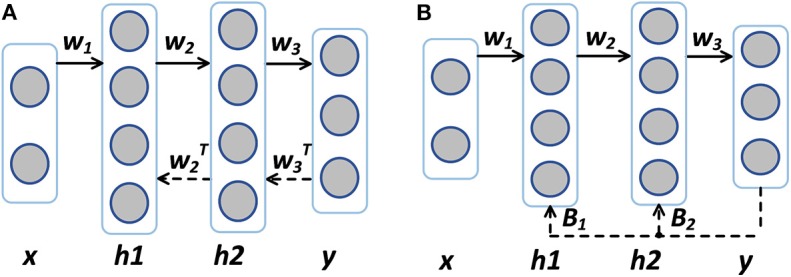
**(A)** Backpropagation (BP) vs. **(B)** Direct feedback alignment (DFA). Solid arrows indicate feedforward paths and dashed arrows indicate feedback paths. The feedback matrices ***B***_1_ and ***B***_2_ need not be symmetric to ***W***_2_ or ***W***_3_.

A more disruptive approach called Direct Feedback Alignment (DFA) is proposed in DNNs (Nøkland, 2016). DFA is compared with BP in Figure 1. Unlike propagating the error back layer by layer in BP and FA, DFA feeds back the error through fixed random feedback connections directly from the output layer to each hidden layer, eliminating the need for layer-by-layer error backpropagation or feedback. DFA is considered more biologically plausible because the error is generated almost completely local with no long backpropagation/feed back train and symmetric weights are not required. Nøkland (2016) shows that for conventional multi-layer ANNs like DNNs, the use of DFA can achieve competitive results with insignificant performance drops when compared with the state-of-the-art BP methods.

In this paper, we extend the DFA for conventional ANNs (Nøkland, 2016) for SNNs. To the best of our knowledge, this is the first work applying DFA to SNNs. Furthermore, our DFA approach, dubbed ST-DFA, operates on the spike-train level, hence offering improved scalability in both space (network depth) and time.

#### 2.1.2. Spike-Train Level Post-synaptic Potential

Before describing the proposed ST-DFA in section 2.2, we present the concept of Spike-train Level Post-synaptic Potential (S-PSP) that is behind the spike-train level computation of ST-DFA.

The widely adopted leaky integrate-and-fire (LIF) model for spiking neurons is given by (Gerstner and Kistler, 2002):

(1) $$\begin{array}{l} \tau_{m}\frac{u_{i}\left(t\right)}{dt}=-u_{i}\left(t\right)+R\;\alpha_{i}\left(t\right), \end{array}$$

with

(2) $$\begin{array}{l} \tau_{s}\frac{\alpha_{i}\left(t\right)}{dt}=-\alpha_{i}\left(t\right)+\underset{j}{\sum }w_{ij}\underset{t_{j}^{\left(f\right)}}{\sum }D\left(t-t_{j}^{\left(f\right)}\right), \end{array}$$

where *u*_*i*_(*t*) is the membrane potential of the neuron *i*, α_*i*_(*t*) is the first order synaptic model with time constant τ_*s*_, and τ_*m*_ is the time constant of membrane potential with value τ_*m*_ = *RC*. *R* and *C* are the effective leaky resistance and effective membrane capacitance. *w*_*ij*_ is the weight of the synapse from the pre-synaptic neuron *j* to the neuron *i*. $t_{j}^{\left(f\right)}$ denotes a particular firing time of the neuron *j*. *D*(*t*) is the Dirac delta function. R is set to 1 since it can be absorbed into synaptic weights.

Integrating (1) and (2) gives the spike response model (SRM) (Jin et al., 2018):

(3) $$\begin{array}{l} u_{i}\left(t\right)=\underset{j}{\sum }w_{ij}\underset{t_{j}^{\left(f\right)}}{\sum }\epsilon \left(t-{\hat{t}}_{i}^{\left(f\right)},t-t_{j}^{\left(f\right)}\right), \end{array}$$

where ${\hat{t}}_{i}^{\left(f\right)}$ denotes the last firing time of the neuron *i*. ϵ(*s, t*) specifies the normalized time course of the *post-synaptic potential* evoked by a single firing spike of the pre-synaptic neuron:

(4) $$\begin{array}{l} \epsilon \left(s,t\right)=\;\frac{1}{C}\int_{0}^{s}\exp\left(-\frac{t^{\prime }}{\tau_{m}}\right)\;\alpha_{i}\left(t-t^{\prime }\right)\text{ d}t^{\prime }. \end{array}$$

Integrating (4) gives:

(5) $$\begin{array}{l} \epsilon \left(s,t\right)=\frac{e^{\left(-\max\left(t-s,0\right)/\tau_{s}\right)}}{1-\frac{\tau_{s}}{\tau_{m}}}\;\left[e^{\left(-\frac{\min\left(s,t\right)}{\tau_{m}}\right)}-e^{\left(-\frac{\min\left(s,t\right)}{\tau_{s}}\right)}\right]H\left(s\right)H\left(t\right), \end{array}$$

where *H*(*t*) is the Heaviside step function.

The sum of the (normalized) post-synaptic potential of the neuron *i* evaluated right before all the neuron *i*'s firing times evoked by the spike train of the pre-synaptic neuron *j* defines the (normalized) spike-train level post-synaptic potential (S-PSP) *e*_*i*|*j*_, which is given by:

(6) $$\begin{array}{l} e_{i|j}=\underset{t_{i}^{\left(f\right)}}{\sum }\underset{t_{j}^{\left(f\right)}}{\sum }\epsilon \left(t_{i}^{\left(f\right)}-{\hat{t}}_{i}^{\left(f\right)},t_{i}^{\left(f\right)}-t_{j}^{\left(f\right)}\right). \end{array}$$

S-PSP specifies the aggregated effect of the spike train of the pre-synaptic neuron *j* on the membrane potential of the post-synaptic neuron *i*, providing a basis for relating firing counts to spike events.

Summing the weighted S-PSPs from all pre-synaptic neurons of the neuron *i* gives the total post-synaptic potential (T-PSP) *a*_*i*_, which is directly correlated to the neuron *i*'s firing count *o*_*i*_ through the firing threshold voltage ν:

(7) $$\begin{array}{l} a_{i}=\underset{j}{\sum }w_{ij}e_{i|j}.\;o_{i}=g\left(a_{i}\right)\approx \frac{a_{i}}{\nu } \end{array}$$

### 2.2. Proposed Spike-Train Level Direct Feedback Alignment (ST-DFA)

#### 2.2.1. Proposed ST-DFA Algorithm

For a conventional (non-spiking) ANN, the squared error for one training example can be defined at the output layer by:

(8) $$\begin{array}{l} E=\frac{1}{2}{\left\|\text{o}-\text{y}\right\|}_{2}^{2}, \end{array}$$

where ***y*** and ***o*** are vectors specifying the desired output (label) and the actual output, respectively. The output *o*_*i*_ of each neuron *i* is determined by the activation function ϕ_*i*_: $o_{i}=\phi_{i}\left(\underset{j}{\sum }w_{ij}x_{j}\right)$, where *x*_*j*_ is the input value from the presynaptic neuron *j* and *w*_*ij*_ is the weight between the neurons *j* and *i*.

The well-known BP algorithm for an ANN (Rumelhart et al., 1988b), which is ubiquitously used in deep learning, is:

(9) $$\begin{array}{l} \Delta w_{ij}=\eta \frac{\partial E}{\partial w_{ij}^{k}}=\eta \delta_{i}^{k}\phi_{j}^{k-1}\\ \delta_{i}^{k}=\{\begin{array}{ll} o_{i}-y_{i} & \text{for output layer},\\ {\phi^{\prime }}_{i}^{k+1}\sum_{l=1}^{r^{k+1}}\delta_{l}^{k+1}w_{li}^{k+1} & \text{for hidden layers}, \end{array} \end{array}$$

where η is the learning rate, $\delta_{i}^{k}$ the error for the *i*th neuron of the *k*th layer, *r*^*k*^ the number of neurons in the *k*th layer.

It has been demonstrated recently that training SNNs using BP with respect to a rate-coded loss function has produced highly competitive performances (Lee et al., 2016; Jin et al., 2018; Wu et al., 2018). Rate-coded loss functions are also adopted for our ST-DFA. Different from BP, the proposed ST-DFA algorithm for SSNs computes each error δ by direct feedback from the output layer on the spike-train level, giving to the following update rule:

(10) $$\begin{array}{l} \Delta w_{ij}=\eta \frac{\partial E}{\partial w_{ij}}=\eta \delta_{i}^{k}e_{i|j}^{k},\\ \;\delta_{i}^{k}=\{\begin{array}{ll} \frac{o_{i}^{o}-y_{i}^{o}}{\nu } & \text{for output layer},\\ \sum_{l=1}^{r^{o}}\delta_{l}^{o}b_{li}^{k} & \text{for hidden layers}, \end{array} \end{array}$$

where η is the learning rate, $\delta_{i}^{k}$ the error of the neuron *i* in the *k*th hidden layer, $e_{i|j}^{k}$ the S-PSP from the neuron *i* to neuron *j*, $o_{i}^{o}$ the actual firing count of neuron *i* in the output layer, $y_{i}^{o}$ the desired firing count for the neuron *i*, ν the firing threshold, *r*^*o*^ the number of neurons in the output layer, $\delta_{l}^{o}$ the error of the neuron *l* in the output layer, and $b_{li}^{k}$ the value of the fixed random feedback.

**Figure 2:**
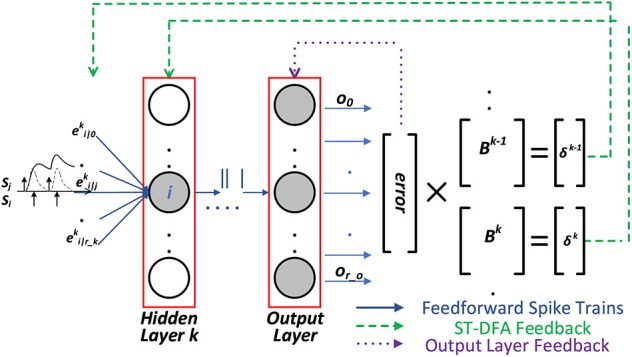
The proposed spike-train level DFA (ST-DFA).

The last equation of (10) is based on the concept of DFA. As in Figure 2, with ST-DFA, the output layer is fully connected to each hidden layer through a different matrix which is called the random feedback matrix **B**. The weights (values) in these matrices are randomly generated and then stay fixed. The error vector ***δ***^*k*^ of the hidden layer *k* is directly obtained from the error vector of the output layer ***δ***^*o*^ and the random feedback matrix ***B***^*k*^ as: ***δ***^*k*^ = ***B***^*k*^ × ***δ***^*o*^. The detailed derivation of ST-DFA is introduced next.

#### 2.2.2. Derivation of ST-DFA

Similar to (8) and using (7), we define the rate-coded loss function as:

(11) $$\begin{array}{l} E=\frac{1}{2}{\left\|\text{o}-\text{y}\right\|}_{2}^{2}=\frac{1}{2}{\left\|\frac{\text{a}}{\nu }-\text{y}\right\|}_{2}^{2}, \end{array}$$

where ***y***, ***o***, and ***a*** are vectors specifying the desired firing counts (label), the actual firing counts, and the T-PSP of the output neurons, respectively. Differentiating the loss function with respect to each trainable weight *w*_*ij*_ leads to:

(12) $$\begin{array}{l} \frac{\partial E}{\partial w_{ij}}=\frac{\partial E}{\partial a_{i}^{k}}\frac{\partial a_{i}^{k}}{\partial w_{ij}}=\delta_{i}^{k}\frac{\partial a_{i}^{k}}{\partial w_{ij}}, \end{array}$$

where $a_{i}^{k}$ is the T-PSP of the neuron *i* in the *k*th layer.

It is instrumental to note that each S-PSP *e*_*i*|*j*_ depends on both rate and temporal information of the pre/post-spike trains, i.e., *e*_*i*|*j*_ depends on the pre/post-synaptic firing counts *o*_*i*_ and *o*_*j*_ and pre/post-synaptic firing times ${\text{t}}_{j}^{\left(f\right)}$ and ${\text{t}}_{i}^{\left(f\right)}$:

(13) $$\begin{array}{l} e_{i|j}=f\left(o_{j},o_{i},{\text{t}}_{j}^{\left(f\right)},{\text{t}}_{i}^{\left(f\right)}\right). \end{array}$$

For the *i*th output neuron, $\delta_{i}^{o}$ can be obtained from (12) and (7):

(14) $$\begin{array}{l} \delta_{i}^{o}=\frac{\partial E}{\partial a_{i}^{o}}=\left(o_{i}-y_{i}\right)\;\frac{\partial o_{i}}{\partial a_{i}}=\frac{o_{i}-y_{i}}{\nu }. \end{array}$$

For each *i*th neuron in the hidden layer *k*, $\delta_{i}^{k}$ is derived from the chain rule based on (7):

(15) $$\begin{array}{l} \delta_{i}^{k}=\frac{\partial E}{\partial a_{i}^{k}}=\overset{r^{k+1}}{\underset{l=1}{\sum }}\;\frac{\partial E}{\partial a_{l}^{k+1}}\;\frac{\partial a_{l}^{k+1}}{\partial a_{i}^{k}}=\overset{r^{k+1}}{\underset{l=1}{\sum }}\delta_{l}^{k+1}\;\frac{\partial a_{l}^{k+1}}{\partial a_{i}^{k}}\\ \;=\overset{r^{k+1}}{\underset{l=1}{\sum }}\delta_{l}^{k+1}w_{li}^{k+1}\;\frac{\partial e_{l|i}^{k+1}}{\partial a_{i}^{k}}. \end{array}$$

The first key development in ST-DFA is that the way in which the error $\delta_{i}^{k}$ is calculated in each hidden layer changes from $\overset{r^{k+1}}{\underset{l=1}{\sum }}\delta_{l}^{k+1}w_{li}^{k+1}\;\frac{\partial e_{l|i}^{k+1}}{\partial a_{i}^{k}}$ to $\overset{r^{o}}{\underset{l=1}{\sum }}\delta_{l}^{o}d_{li}^{k}\;\frac{\partial e_{l|i}^{k+1}}{\partial a_{i}^{k}}$, where $d_{li}^{k}$ is the direct feedback alignment from the output neuron *l* to the hidden layer neuron *i*. $d_{li}^{k}$ is a randomized and fixed value. In this process, we replace the $w_{li}^{k+1}$ from (*k* + 1)th layer to *k*th layer in (15) by $d_{li}^{k}$, leading to:

(16) $$\begin{array}{l} \delta_{i}^{k}=\delta_{l}^{o}d_{li}^{k}\;\frac{\partial e_{l|i}^{k+1}}{\partial a_{i}^{k}}. \end{array}$$

As such, the error ***δ***^*k*^ of each hidden neuron is directly determined by the output layer error vector ***δ***^*o*^ rather than by the error vector ***δ***^*k*+1^ of the next layer.

Moreover, we have the following key observation. In (16), since $d_{li}^{k}$ is randomly generated, $\frac{\partial e_{l|i}^{k+1}}{\partial a_{i}^{k}}$ can be absorbed into $d_{li}^{k}$ to further simplify ST-DFA. Denote the new DFA parameter absorbing $\frac{\partial e_{l|i}^{k+1}}{\partial a_{i}^{k}}$ by $b_{li}^{k}=d_{li}^{k}\frac{\partial e_{l|i}^{k+1}}{\partial a_{i}^{k}}$, the simplified error computation becomes:

(17) $$\begin{array}{l} \delta_{i}^{k}=\{\begin{array}{ll} \frac{o_{i}^{o}-y_{i}^{o}}{\nu } & \text{for output layer},\\ \sum_{l=1}^{r^{o}}\delta_{l}^{o}b_{li}^{k} & \text{for hidden layers}, \end{array} \end{array}$$

where $b_{li}^{k}$ is one entry of the random feedback matrix ***B*** in Figure 2.

Thus, ST-DFA reduces the computational complexity by not only avoiding layer-by-layer propagation but also the additional simplification via the use of $b_{li}^{k}$.

#### 2.2.3. Simplification for Hardware Friendliness

The last term on the right-hand side of (12) differentiates the total post-synaptic potential (T-PSP) $a_{i}^{k}$. Considering (7), it can be written as:

(18) $$\begin{array}{l} \begin{array}{l} \frac{\partial a_{i}^{k}}{\partial w_{ij}}=\frac{\partial }{\partial w_{ij}}\left(\overset{r^{k-1}}{\underset{j=1}{\sum }}w_{ij}e_{i|j}^{k}\right)=e_{i|j}^{k}+\overset{r^{k-1}}{\underset{l=1}{\sum }}w_{il}\frac{\partial e_{i|l}^{k}}{\partial o_{i}^{k}}\;\frac{\partial o_{i}^{k}}{\partial w_{ij}}\\ \;=e_{i|j}^{k}+\frac{e_{i|j}^{k}}{\nu }\;\overset{r^{k-1}}{\underset{l=1}{\sum }}w_{il}\frac{\partial e_{i|l}^{k}}{\partial o_{i}^{k}}. \end{array} \end{array}$$

The exact evaluation of the above expression requires multiple additions, multiplications, and divisions, introducing high hardware overhead and additional latency.

The first term $e_{i|j}^{k}$ on the right-hand side of (18) can be interpreted as the direct influence exerted on the T-PSP $a_{i}^{k}$ by changing the synaptic weight *w*_*ij*_ as seen from (7). The second term $\frac{e_{i|j}^{k}}{\nu }\;\overset{r^{k-1}}{\underset{l=1}{\sum }}w_{il}\frac{\partial e_{i|l}^{k}}{\partial o_{i}^{k}}$ comes from the fact that changing the weight *w*_*ij*_ leads to variation in the post-synaptic spike train. Thus, the S-PSP $e_{i|l}^{k}$ to the neuron *i* also varies as it depends on the firing times of the post-synaptic neuron. Nevertheless, we have observed that the first term dominates the second term. By dropping the second term, we reach the final hardware-friendly ST-DFA algorithm of (10), which also maintains good performance.

In comparison, the spike-train level BP algorithm HM2-BP is (Jin et al., 2018):

(19) $$\begin{array}{l} \Delta w_{ij}=\eta \delta_{i}^{k}e_{i|j}^{k}\left(1+\frac{1}{\nu }\;\sum_{l=1}^{r^{k-1}}w_{il}\frac{\partial e_{i|l}^{k}}{\partial o_{i}^{k}}\right),\\ \delta_{i}^{k}=\{\begin{array}{ll} \frac{o_{i}^{k}-y_{i}^{k}}{\nu } & \text{for output layer},\\ \frac{1}{\nu }\sum_{l=1}^{r^{k+1}}\delta_{l}^{k+1}w_{li}\frac{\partial e_{l|i}^{k+1}}{\partial o_{i}^{k}} & \text{for hidden layers}. \end{array} \end{array}$$

While HM2-BP delivers the state-of-the-art performance, it would be very costly to implement on hardware if ever feasible.

In all, compared to HM2-BP in (19), ST-DFA in (10) is much more hardware friendly. With ST-DFA, direct error feedback to each hidden layer is accomplished without layer-by-layer back propagation while HM2 requires high-resolution multiplications with the transpose of the forward weights and other expensive operations layer by layer. In the next section, we efficiently realize the ST-DFA algorithm on digital hardware.

### 2.3. SNN Accelerators With ST-DFA On-Chip Training

#### 2.3.1. Architecture

**Figure 3:**
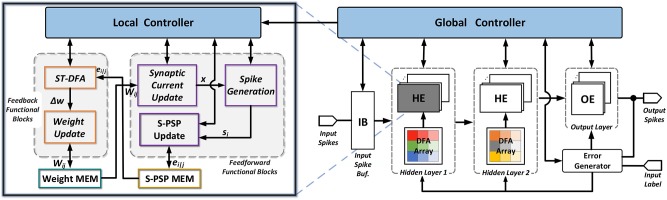
Proposed architecture of multi-layer SNNs with onchip ST-DFA training. HE represents a digital hidden neuron element; and OE represents a digital output neuron element.

Figure 3 shows the architecture of the proposed multi-layer feed-forward spiking neural processors with the proposed ST-DFA on-chip training. Only two hidden layers are shown for illustration purpose. Architecturally, the processor is comprised of an input spike buffer feeding multiple hidden layers composed of hidden neuron elements (HEs). The last hidden layer connects to the output layer which consists of a set of output neuron elements (OEs). A modular design approach is taken where each spiking neuron is implemented in the form of HE or OE. As such, a proper number of HEs and OEs can be instantiated to form a multi-layer SNN with arbitrary depth and width.

Both inference and training are supported. Training over an input example splits into two phases: forward pass and backward pass. The computation of S-PSPs required for ST-DFA training are computed in an online manner in the forward pass of training. The remaining computations of the forward pass are identical to those performed in inference. To support ST-DFA training, the error generator utilizes an array of subtractors to compute the difference between the actual OE output spike counts with expected ones (label). At each hidden layer, this output-layer error vector is multiplied with the associated ST-DFA random feedback matrix inside each layer to allow weight updates performed by each neuron.

- On-chip training

For each training example, the forward and backward passes of the training are controlled by a global controller (FSM) as shown in Figure 3. Neurons at the same layer process information in parallel to exploit the inherent parallelism of the hardware SNN processor architecture. In the forward pass and at each biological time step, layers are activated by the global controller one at a time from the input to the output. After output spikes are generated for the current time step, the global controller pushes the training forward to the next time step. This process repeats until the current training example has been entirely learned by the network. Then, the backward pass starts, in which the first step is to calculate the output error $\delta_{l}^{o}$ in (10). After that, all hidden layers start to perform ST-DFA for weight updating at the same time. The weight update latency of each hidden layer may be different due to the differences in the number of input synaptic connections (i.e., the preceding layer width). After all hidden layers finish ST-DFA weight updates, the training process moves onto the next training example.

- Neuron unit design

Each HE or OE contains several functional blocks categorized into feed-forward functional blocks and feedback functional blocks as shown in Figure 3. OEs are identical to HEs except that no ST-DFA module is included since the error $\delta_{i}^{k}$ defined for output neurons is computed by the Error Generator module. Each neuron unit contains two memory modules that store the synaptic weights and all its spike-train level post-synaptic potentials (S-PSPs), respectively. We implement the weight memory with block RAM (BRAM) and the S-PSP memory with a 2-D array of flip flops (FFs) on the FPGA. A neuron-level local controller (FSM) controls the detailed inference/training steps. The local controller also communicates with the global controller for synchronizing processes between different layers and inference/training stages.

In the forward pass of training, first, the synaptic current *x* through each synapse is calculated, followed by the spike-train level post-synaptic potential (S-PSP) update for the same synapse. The synaptic current update and the S-PSP update modules shown in Figure 3 are shared by all input synapses. Hence, processing of all synapses are done in series. After all synaptic responses are generated, the spike generation module calculates the neuron's membrane potential and makes the firing decision based on the leaky integrate-and-fire (LIF) spiking neuron model. In the backward pass of training, the ST-DFA module implements the proposed on-chip ST-DFA training algorithm, the output of which is then fed to the weight update module. Finally, the corresponding synaptic weight is updated and stored back to the weight memory. Similar to the feedforward blocks, the feedback functional modules are also shared among all input synapses.

#### 2.3.2. Efficient On-Chip S-PSP Calculation

One important component in the proposed ST-DFA algorithm is the spike-train level post-synaptic potential (S-PSP), *e*_*i*|*j*_, in (10). As demonstrated in (6), by definition, *e*_*i*|*j*_ is the effect of all firing events of the pre-synaptic neuron *j* on the post-synaptic neuron *i*. However, direct implementation of (6) on hardware is very costly; all firing events of the pre- and post-synaptic neurons need to be stored and excessive multiplication, division and exponentiation operations are involved, incurring much logic complexity and memory usage.

Instead, we propose an online S-PSP calculation approach with dramatically reduced hardware overhead. Rather than recording all firing events of the two neurons and computing *e*_*i*|*j*_ at once in the backward pass, in the forward pass we accumulate and update *e*_*i*|*j*_ at the arrival of each firing event and store the updated *e*_*i*|*j*_ in the S-PSP memory of each neuron element.

Inspecting (3) and (6) reveals that *e*_*i*|*j*_ is the normalized (by weight) of the contribution from the post-synaptic neuron *j* to the aggregated membrane potential of the post-synaptic neuron *i*. While the aggregated post-synaptic membrane potential is effectively tracked by the LIF model, each individual contribution *e*_*i*|*j*_ to it can be accumulated exactly using the following equations:

(20) $$\begin{array}{l} \begin{array}{l} \tau_{s}\frac{p_{i|j}\left(t\right)}{dt}=-p_{i|j}\left(t\right)+\underset{t_{j}^{\left(f\right)}}{\sum }D\left(t-t_{j}^{\left(f\right)}\right),\\ \tau_{m}\frac{q_{i|j}\left(t\right)}{dt}=-q_{i|j}\left(t\right)+p_{i|j}\left(t\right),\\ e_{i|j}\left(t\right)=\underset{t_{i}^{\left(f\right)}}{\sum }q_{i|j}\left(t_{i}^{\left(f\right)}\right), \end{array} \end{array}$$

where *p*_*i*|*j*_(*t*) is the (normalized) synaptic input from the neuron *j* to neuron *i*, which is part of (2), and *q*_*i*|*j*_(*t*) is interpreted as the (normalized) post-synaptic membrane voltage contribution from the neuron *j* to neuron *i*, which shall be reset to zero when the neuron *i* fires at a particular firing time $t_{i}^{\left(f\right)}$.

The hardware realization of (20) is based on discretizing it using the first-order Euler method with a fixed stepsize:

(21) $$\begin{array}{l} q_{i|j}[t+1]=(1-\frac{1}{\tau_{m}})q_{i|j}[t]+p_{i|j}[t+1]\\ p_{i|j}[t+1]=(1-\frac{1}{\tau_{s}})p_{i|j}[t]+\frac{1}{\tau_{s}}\sum_{t_{j}^{\left(f\right)}}D_{n}(t-t_{j}^{\left(f\right)})\\ \{\begin{array}{l} e_{i|j}[t+1]+=q_{i|j}[t+1]\\ q_{i|j}[t+1]=0 \end{array}\;if\;t+1=t_{i}^{\left(f\right)}, \end{array}$$

where *D*_*n*_(·) is the unit sample function and we have abused the notation by using *t* and *t* + 1 to indicate a discrete time step and the step after that.

**Figure 4:**
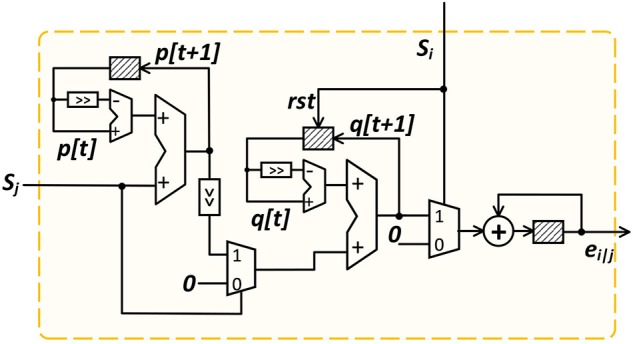
On-line S-PSP calculation onchip.

(21) allows *e*_*i*|*j*_ to be accumulated in an online manner with great hardware efficiency and its implementation is shown in Figure 4. At each time step, we first update the value of *p*_*i*|*j*_, followed by the updates of *q*_*i*|*j*_ and *e*_*i*|*j*_, controlled by the FSM states of the local controller shown in Figure 3. The shaded blocks in Figure 4 are registers used to store the current-time variable values. We set both decay constants τ_*s*_ and τ_*m*_ to be a power of 2 such that multiplications/divisions are realized efficiently using shift operations. The updated *e*_*i*|*j*_ is stored in the S-PSP memory and retrieved by the ST-DFA module during the backward training pass.

#### 2.3.3. Efficient On-Chip ST-DFA Implementation

**Figure 5:**
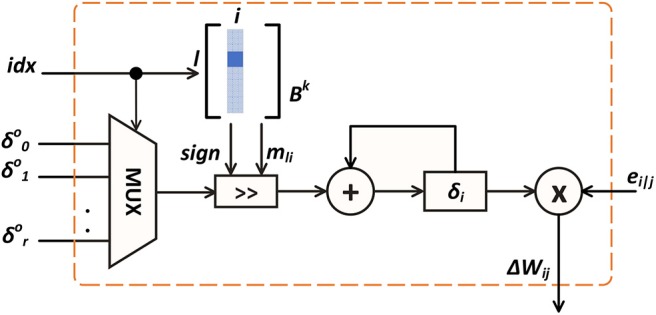
On-chip ST-DFA weight update computation.

Figure 5 depicts the ST-DFA module in hidden neurons shown in Figure 3. As in (10), for each hidden neuron *i*, the inner product between the error vector $\delta_{l}^{o}$ from the output layer and the *i*th column of the random feedback matrix *B* of the corresponding layer is computed. The inner product is then multiplied with *e*_*i*|*j*_ to produce the weight update value Δ*w*_*ij*_ for the *j*th input synapse. All these inner products for different synapses are computed in series and would result in large hardware and power overheads. Furthermore, if each entry of the feedback matrix is set to be a high-bit resolution random number, high memory usage is required for storage.

To mitigate the above design complexity, we propose a hardware-friendly realization of ST-DFA, named ST-DFA-2. ST-DFA-2 is based on the key observation from extensive algorithmic experiments that the feedback matrix *B* need not be generated in a true random manner; setting each entry *b*_*li*_ of *B* to one of a small set of fixed numbers at random is sufficient for achieving good training performance. Furthermore, the set of fixed numbers can be optimized for hardware efficiency. For this, we construct this set by making each number a signed power of 2 with low-bit resolution such that the multiplications in (10) can be implemented by shift operations and storage for *B* is kept at minimal.

Figure 5 illustrates the computation of each weight update. The corresponding inner product is computed by accumulating the element-wise products. The *idx* signal selects a particular element in the error vector $\delta_{l}^{o}$ and its shift amount *m*_*il*_, which is set by the corresponding *b*_*li*_ in the *B* matrix according to $|b_{li}|=2^{m_{il}}$. If *b*_*li*_ is negative, the shift result is converted to its compliment before added to δ_*i*_. Finally, the resulting δ_*i*_ is multiplied with the S-PSP *e*_*i*|*j*_ to get the weight update value Δ*w*_*ij*_ for the current synapse.

## 3. Results

### 3.1. Experimental Settings and Benchmarks

Performance evaluation is divided into two parts:

1. Section 3.2 devotes to evaluate the performance of proposed ST-DFA and ST-DFA-2 compared to HM2-BP only with software simulation. The classification performances are evaluated by simulation of the digital computations with the actual bit resolutions implemented on FPGA. Major SNN variables, for example synaptic weight *w*, S-PSP *e*_*i*|*j*_ and membrane potential *v*, are in the fixed-point representation. Each *w* is a signed 17-bit variable with 12-bit fractional. 11 bits are used for each unsigned variable *e*_*i*|*j*_ with 6-bit fractional and 9 bits are used for each signed variable *v* with 3-bit fractional.
2. In section 3.3, we measure various aspects of the SNN neural processor based on the pure hardware platform (on-board measurement). We measure the performance vs. hardware overhead tradeoffs of the proposed on-chip ST-DFA training on several feed-forward SNN neural processors. Using multiple SNNs models with varying depths and widths, we demonstrate competitive performance of pure hardware (on-board) simulation. Compared to hardware implementation of HM2-BP, proposed ST-DFA significantly reduces hardware overhead which proves hardware-friendliness. FPGA prototypes of SNN neural accelerators are designed on the Xilinx ZC706 platform for performance evaluation, design overhead, and power/energy analysis.

Three datasets are employed for evaluation: MNIST (LeCun et al., 1998), N-MNIST, or the neuromorphic version of MNIST (Orchard et al., 2015), and the 16-speaker English letter subset of the TI46 speech corpus (Liberman et al., 1991). The MNIST handwritten digit dataset (LeCun et al., 1998) contains 60k training and 10k testing examples, each of which is a 28 × 28 grayscale image. Each pixel value of the MNIST image is converted into a spike train using Poisson sampling and the probability of spike generation is proportional to the pixel intensity. Due to the limited hardware resources available on the Xilinx Zynq ZC706 board, we crop each image to include only the 14 × 14 pixels around the center for FPGA evaluation.

The N-MNIST dataset (Orchard et al., 2015) is a neuromorphic version of MNIST. The static digit images of MNIST are converted into spike trains using a dynamic vision sensor (DVS) (Lichtsteiner et al., 2008) moving on a pan-tilt unit. The image is resized to 34 × 34 since the relative shift of images during the saccade process is required. Two kinds of spike events, ON and OFF, are recorded since the intensity can either increase or decrease. Thus, each N-MNIST image has 34 × 34 × 2 = 2, 312 spike sequences lasting for about 300 ms. We reduce the time resolution of the N-MNIST images by 500 × to speed up the processing.

The TI46 Speech corpus (Liberman et al., 1991) contains spoken English letters from 16 speaker. There are 4,142 and 6,628 spoken English letters for training and testing, respectively. The continuous temporal speech waveforms are first preprocessed by the Lyon's ear model (Lyon, 1982) and then encoded into 78 spike trains using the BSA algorithm (Schrauwen and Van Campenhout, 2003).

Among these datasets, MNIST and TI46 are tested on both software and hardware while N-MNIST is only tested on software simulation due to that the available FPGA resources are not sufficient to support the large number of spike trains. Moreover, to thoroughly assess the classification performance and hardware benefits of our proposed spike-train level direct feedback alignment (ST-DFA), we build multiple SNNs with different network depths and widths.

### 3.2. Classification Accuracies (Software Simulation)

The proposed spike-train level direct feedback alignment (ST-DFA) algorithm is inspired by the spike-train level backpropagation HM2-BP algorithm. In Jin et al. (2018), HM2-BP is compared with other state-of-the-art spiking or non-spiking BP methods, such as spike-based BP (Lee et al., 2016), STBP (Wu et al., 2018), temporal coding BP (Mostafa, 2018), and non-spiking BP (Neil et al., 2016) on MNIST and N-MNIST. Apart from its high efficiency due to the spike-train level processing, HM2-BP outperforms or is on a par with all these recently developed algorithms. For example, with a single hidden layer of 800 neurons, HM2-BP can achieve 98.93% accuracy on MNIST while Neil et al. (2016) gets up to 98.30%. HM2-BP obtains 98.88% accuracy on N-MNIST compared with 97.80% by Mostafa (2018). Moreover, HM2-BP delivers competitive performance on challenging benchmarks, such as the 16-speaker spoken English letters of TI46 Speech corpus (Liberman et al., 1991) and 47-class image recognition dataset Extended MNIST (EMNIST) (Cohen et al., 2017).

As presented in section 2.2, ST-DFA propagates the errors δ from the output layer to each hidden layer directly without layer by layer error backpropagation through symmetric weights matrices. In section 2.3.3, we further optimize ST-DFA by setting each entry of the random feedback matrix ***B*** to a power of 2, leading to the hardware-friendly ST-DFA-2 algorithm. In this work, feedback matrix entries are randomly chosen from the set {−4, −2, −1, 0, 1, 2, 4} for ST-DFA-2.

Table 1 compares the inference accuracies of HM2-BP, ST-DFA, and ST-DFA-2 on MNIST, N-MNIST, and TI46. Compared to HM2-BP, ST-DFA, and ST-DFA-2 still maintain rather competitive performance while the low computational cost and hardware-friendliness of ST-DFA-2 translate into huge hardware resources and energy overhead savings as shown later. It shall be noted that in comparison with ST-DFA, ST-DFA-2 does not necessarily degrade performance; it can even slightly outperform ST-DFA in practice.

**Table 1:** Inference accuracy comparison of HM2BP, ST-DFA, and ST-DFA-2 derived by software simulation.

**Dataset**	**Learning rule and network structure**	**Accuracy (%)**
MNIST	HM2-BP: 784-800-10	98.93
MNIST	ST-DFA: 784-800-10	98.64
MNIST	ST-DFA-2: 784-800-10	98.74
N-MNIST	HM2-BP: 2312-800-10	98.88
N-MNIST	ST-DFA: 2312-800-10	98.47
N-MNIST	ST-DFA-2: 2312-800-10	98.59
TI46	HM2-BP: 78-800-26	89.92
TI46	ST-DFA: 78-800-26	87.00
TI46	ST-DFA-2: 78-800-26	87.31

*All SNNs are fully connected networks with a single hidden layer of 800 neurons. MNIST: 28 × 28 input resolution; N-MNIST: 2,312 input spike trains; 16-speaker TI46: 78 input spike trains*.

### 3.3. FPGA Hardware Evaluations (On-Board Measurement)

We build several FPGA SNN accelerators on the targeted Xilinx ZC706 platform, the sizes of which are decided considering the available resources on-chip. Tables 2, 3 shows the resource and energy overhead as well as the inference accuracies of these SNN accelerators with on-chip ST-DFA-2. Training powers are estimated by the Xilinx Power Analyzer based on application-specific workloads. With the result of behavioral simulation using a binary-converted input data sample, the tool measures the dynamic power of neural processors clocked at 100MHz as presented in Table 2. Compared hardware cost in two different designs, i.e., ST-DFA-2 and HM2-BP, is shown in Table 4.

**Table 2:** Overheads of the fully-connected SNNs with on-chip ST-DFA-2 implemented on Xilinx ZC706 board.

**MNIST (14** **×** **14 input resolution) @100 MHz**						
	**Resource utilization**			**Training power (mW)**	**Training latency (mS)**	**Training energy (mJ)**
	**LUTs**	**FFs**	**DSPs**			
196-50-10	33484	6836	60	113	3.998	0.452
196-50-50-10	62989	12516	110	125	4.836	0.604
196-100-10	73027	12329	110	224	4.802	1.076
196-100-100-10	126482	23331	210	275	6.445	1.772
**TI46 (16-speaker Spoken English Letters) @100 MHz**						
	**Resource utilization**			**Training power (mW)**	**Training latency (mS)**	**Training energy (mJ)**
	**LUTs**	**FFs**	**DSPs**			
78-50-26	38220	8826	76	73	3.688	0.269
78-50-50-26	74709	14641	126	87	5.123	0.445
78-100-26	64280	14096	126	113	5.089	0.575
78-100-100-26	145452	30546	226	185	7.929	1.467

**Table 3:** Inference performances of the fully-connected SNNs with on-chip ST-DFA-2 measured on Xilinx ZC706 FPGA board.

**MNIST (14** **×** **14 resolution) @100 MHz**	
	**Accuracy (On-board) (%)**
196-50-10	94.34
196-50-50-10	94.51
196-100-10	95.72
196-100-100-10	96.27
**TI46 (16-speaker English Letters) @100 MHz**	
	**Accuracy (On-board) (%)**
78-50-26	71.63
78-50-50-26	74.95
78-100-26	75.19
78-100-100-26	84.88

**Table 4:** Overheads of an FPGA SNN with on-chip HM2-BP vs. ST-DFA-2 (Network size:196-100-100-10).

	**LUTs**	**FFs**	**DSPs**	**Backward phase latency (uS)**
HM2-BP	154477	23462	900	17.560
ST-DFA	126482	23331	210	12.010
	**Normalized LUTs (%)**	**Normalized FFs (%)**	**Normalized DSPs (%)**	**Normalized B-P latency (%)**
HM2-BP	122	101	429	146
ST-DFA	100	100	100	100

As shown in Tables 2, 3, the implemented networks have either one or two hidden layer(s), and each hidden layer has 50 or 100 neurons. Numbers of input and output neurons are application-dependent. The training latency and training energy are for training a representative input example of the corresponding dataset using one iteration of forward and backward passes. Table 2 indicates that the SNNs integrated with ST-DFA-2 in general have efficient FPGA resource utilization as well as low training energy dissipation.

Furthermore, with a trimmed down input size and/or constrained network size, the FPGA SNNs with on-chip ST-DFA-2 can still deliver competitive classification performance in reference to the simulated accuracies achieved at full input size and by larger networks reported in Table 1. For instance, the accuracy of MNIST in Table 1 is based on full input resolution which is 28 × 28 with a hidden layer of 800 neurons. However, we implemented on-board simulation with reduced input resolution and smaller networks due to the Xilinx ZC706 board resource limitation. We cropped each data of MNIST into 14 × 14 which causes 4X input resolution reduction and built smaller networks consists of 50 or 100 hidden neurons as shown in Table 3. Despite the low resolution and the small network size, SNN neural processors with on-chip ST-DFA training show competitive classification accuracy of 96.27% for MNIST, 84.88% for TI46 speech corpus, respectively.

To better illustrate the cost-effectiveness of the proposed ST-DFA algorithm, we also compare the overheads of implementing HM2-BP vs. ST-DFA-2 in a fully-connected SNN FPGA with two hidden layers in Table 4. Since the main difference between HM2-BP and ST-DFA is the backward pass algorithm, we designed HM2-BP in hardware based on the weight updating algorithm represented in Jin et al. (2018). Training latency of the backward pass of the corresponding SNN neural processor is also presented in the table. We do not consider forward pass latency and inference latency since they do not differ significantly in the two cases. The results in the table indicate that ST-DFA is much more efficient in terms of hardware implementation on both resource utilization and backward pass latency compared with HM2-BP. The ST-DFA-2 based SNN neural processor saves 18% on LUTs, 76.7% on DSPs and 31.6% on backward phase latency compared with the HM2-BP based SNN.

## 4. Discussions

While Direct Feedback Alignment (DFA) has been attempted, this work present first novel approach for implementing hardware spiking neural networks (SNNs) on FPGA board. This work aims to build efficient on-chip training FPGA SNN neural processor with reduced backward training latency and hardware cost while gracefully trading off classification performance. To be specific, Table 3 shows competitive software/hardware inference accuracy despite reduce input resolution and small network size. Comparing the accuracy of ST-DFA and ST-DFA-2, performance using ST-DFA can be slightly better than using ST-DFA-2 and vice versa. This fact shows that the error may vary based on randomly initialized feedback weight matrix, which makes ST-DFA-2 still powerful. Table 4 shows the advantages of ST-DFA-2 over HM2-BP in terms of hardware resource utilization through the DFA algorithm and the efficient design of the hardware design units. As shown in Tables 1, 3, this result proves the practicality of the DFA algorithm and the feasibility of implementing the ST-DFA algorithm for on-chip training of the SNN processor.

The large additional hardware overhead and backward latency of HM2-BP mainly come from the layer-by-layer error propagation and the required multiplication operations. Moreover, as the network goes deeper, the backward phase latency grows proportionally in HM2-BP, while in ST-DFA the backward latency will not affect by the network depth since the error processing is concurrently executed in all hidden layers. This property assures the scalability of ST-DFA which is promising for deeper networks. With the proposed ST-DFA algorithm, we have sidestepped the complex backpropagation and enabled cost-effective on-chip training for multi-layer SNNs.

As discussed in section 1, implementing training of SNNs using a BP algorithm suffers from high computing complexity and thus high resource utilization, while a hardware-friendly, non-BP algorithm, such as STDP, suffers from achieving good accuracies. We argue that our approach avoids high computation complexity by extending a BP-like algorithm (i.e., DFA) for SNNs while maintaining the advantage of a well-defined BP-like algorithm in terms of accuracy. To the best of our knowledge, this is the first work presenting algorithm-hardware co-optimization and demonstrating the realization of DFA, which is an efficient on-chip training algorithm, for SNNs.

For example, to compare with existing onchip works, Yin et al. (2017) presented a new BP based training algorithm for discrete-time SNNs by using a LIF neuron model with a gradient estimator. This paper introduced a ReLU-like gradient estimation method to avoid the zero-gradient issue in conventional SNNs using LIF neurons. However, as the experiment results in Yin et al. (2017) are based on off-chip training, we guess that this new BP algorithm still suffers from an efficient on-chip training method. We think that the main difference of Yin et al. (2017) and our work is that Yin et al. (2017) proposed a new BP-based learning algorithm while our work proposes a new BP-like learning algorithm (i.e., DFA) based on a state-of-the-art BP algorithm and efficiently implement it in hardware while delivering competitive accuracy. As a small scale low-power accelerator, Zheng and Pinaki (2018) proposed a hardware-friendly STDP on-chip training algorithm. This paper focuses on capturing the estimated gradients concerning STDP behaviors. By simplifying the calculation of STDP based gradient for weight updating, this work presented an efficient on-chip learning algorithm that can be implemented on hardware. However, this paper still suffers from accuracy, and the main difference is that our work is focusing on BP-like training algorithms which have demonstrated excellent performance in recent years.

However, several challenges should be addressed to achieve more practical application. Although this paper proposes hardware-efficient designs, the resource limitation of the FPGA board does not allow large networks. For instance, we reduced the input resolution of MNIST dataset from 784 to 196 due to the limitation. While the proposed DFA based on-chip learning is demonstrated using relatively small SNNs on FPGA due to hardware resource limitations, our future work will explore a number of techniques, such as more advanced neuron model simplification, architectural level optimization, and/or a larger FPGA board to demonstrate larger-scale SNNs.

Nevertheless, the main focuses of this paper have been on extending the DFA concept proposed by Nøkland (2016) to efficient training of SNNs, and significantly reducing the hardware cost by algorithm-hardware co-optimization while maintaining a competitive accuracy. This paper proposes a novel spike-level direct feedback alignment (ST-DFA) algorithm for training multi-layer spiking neural networks (SNNs) with improved bio-plausibility and scalability over traditional backpropagation algorithms. Moreover, it is demonstrated that the ST-DFA algorithm with its hardware-friendly optimized implementation enable efficient on-chip training of FPGA SNN neural processors while delivering competitive classification performance for practical speech and image recognition tasks.

## Data Availability Statement

The datasets generated for this study are available on request to the corresponding author.

## Author Contributions

JL implemented the on-board simulation and performed experimental studies on the Xilinx ZC706 FPGA board. JL and RZ co-designed the hardware architecture of ST-DFA including all hardware units, such as S-PSP calculation block and ST-DFA weight update block. WZ and PL developed the theoretical approach of Direct Feedback Alignment and provided software simulation. PL defined and directed the overall research. JL, RZ, WZ, YL, and PL wrote the paper. RZ and YL performed this work while being at Texas A&M University.

### Conflict of Interest

The authors declare that the research was conducted in the absence of any commercial or financial relationships that could be construed as a potential conflict of interest.
